# Effectiveness of a SNAPPS in psychiatric residents assessed using objective structured teaching encounters: a case-control study

**DOI:** 10.1590/1516-3180.2021.1028.R1.13072022

**Published:** 2022-10-03

**Authors:** Lorena Pinho Feijó, Guilherme Abreu Pereira, Vitor Maia Teles Ruffini, Fernando Salvetti Valente, Renato Antunes dos Santos, Saadallah Azor Fakhouri, Maria do Patrocínio Tenório Nunes, Kristopherson Lustosa Augusto

**Affiliations:** I MSc. Physician and Assistant Professor, Department of Social Department, Centro Universitário Unichristus, Fortaleza (CE), Brazil.; II MD. Attending Physician, Department of Internal Medicine, Hospital das Clinicas HCFMUSP, Faculdade de Medicina, Universidade de Sao Paulo, Sao Paulo, SP, BR.; III MD. Physician and Associate Professor, Department of Internal Medicine, Universidade Santo Amaro (UNISA), São Paulo (SP), Brazil; and Fellow of Hospital Medicine, Faculdade de Medicina FMUSP, Universidade de Sao Paulo, Sao Paulo, SP, BR.; Universidade de Sao Paulo, Faculdade de Medicina FMUSP, Fellow of Hospital Medicine, Sao Paulo, SP, Brazil; IV MD. Physician and Assistant Professor, Department of Internal Medicine, Faculdade de Medicina FMUSP, Universidade de Sao Paulo, Sao Paulo, SP, BR.; V PhD. Physician and Assistant Professor, Department of Psychiatry, University of Toronto, Toronto, Canada. And Adjunct Professor, Department of Psychiatry, McMaster University, Ontario, Canada; McMaster University, Department of Psychiatry, Ontario, Canada; VI PhD. Physician and Professor, Department of Internal Medicine, Universidade Federal de Uberlândia (UFU), Uberlândia (MG), Brazil.; VII PhD. Physician and Associate Professor, Department of Internal Medicine, Faculdade de Medicina FMUSP, Universidade de Sao Paulo, Sao Paulo, SP, BR.; VIII PhD. Physician and Assistant Professor, Department of Internal Medicine, Faculdade de Medicina, Universidade de Fortaleza (UNIFOR), Fortaleza (CE), Brazil; Postgraduate Professor at master's level, Centro Universitário Christus-Unichristus Fortaleza (CE), Brazil; and Assistant Professor, Department of Internal Medicine, Faculdade de Medicina da Universidade Federal do Ceará (UFC), Fortaleza (CE), Brazil.; Centro Universitário Christus-Unichristus Fortaleza, CE, Brazil; Universidade Federal do Ceará, Faculdade de Medicina, Department of Internal Medicine, Fortaleza, CE, Brazil

**Keywords:** Psychiatry, Internship and residency, Education, medical, Medical education, Medical residency, Active learning

## Abstract

**BACKGROUND:**

Residents play the role of teachers in almost one-quarter of their activities in residency programs.

**OBJECTIVE:**

To evaluate whether a 45-minute class using summarize, narrow, analyze, probe, plan, and select (SNAPPS) could improve psychiatry residents’ case discussion skills in diverse practical learning settings.

**DESIGN AND SETTING:**

This case-control, randomized, blinded study was conducted in a psychiatry hospital at Fortaleza-Ceará.

**METHODS:**

Using “resident as teacher” (RaT), objective structured teaching encounters (OSTEs), and SNAPPS, we conducted a study with 26 psychiatry residents. We analyzed video footage of psychiatric cases in three settings: outpatient, nursing, and emergency. An intervention was held two months later with the residents, who were then assigned to two groups: group A (lecture on SNAPPS) and group B (lecture on a topics in psychiatry). Shortly after the lectures, they were video recorded while discussing the same cases. Three blinded examiners analyzed the videos using an instrument based on the Stanford Faculty Development Program (SFDP-26).

**RESULTS:**

We found high internal consistency among external examiners and an interaction effect, group effect, and moment effect (P < 0.05). The residents who received the SNAPPS lecture scored significantly higher than their counterparts who received a traditional case presentation.

**CONCLUSION:**

This study indicates the efficacy of SNAPPS over traditional case presentation in all three settings as assessed by OSTEs and supports its implementation to improve the teaching of clinical reasoning.

## INTRODUCTION

Residents play the dual role of learners and teachers for up to one-quarter of their time in residency programs.^1^ Nevertheless, formal training in teaching-learning techniques developed for residents, or at the least specific recommendations and regulations for those activities are scant. In addition, teaching skills are difficult to correlate directly with clinical diagnostic and recognized competences. Thus, residents are likely to adopt ineffective teaching strategies.^2^

In the United States, more than 50% of residency programs have already implemented “Resident as Teacher” (RaT) training.^2^ More recently, a study of program directors in the United States showed that RaT has been implemented in 80% of residency programs, representing a 26.34-point increase from 2001 to 2016.^3^ Often, residency programs use a variety of methodologies to teach RaT techniques, including workshops, lectures, seminars, and teaching retreats. These programs have been shown to improve residents' teaching skills^4^ and satisfaction with programs, promoting positive changes in their attitudes toward teaching. A systematic review conducted in 2008 analyzed 13 studies carried out with residents of programs in different fields, demonstrating an improvement in residents' teaching skills in the most diverse techniques employed.^5^ More recently, a review of RaT in general surgery found that changes in attitude toward teaching was the most frequent outcome of assessment,^6^ and a resident-as-teacher consensus guideline has been developed to provide a road map for program directors and institutions and to enhance the culture of teaching and learning.^7^

After the implementation of an RaT curriculum, it might be beneficial to use objective structured teaching encounters (OSTEs) in conjunction with these pedagogical strategies to allow the standardized assessment of skills over time.^8^ OSTEs have proven to be an effective method to assess both residents and medical students,^9^ has and have been used to assess and improve the teaching performance of faculty members.^10^

There are benefits of RaT programs for different participants: Residents, by acquiring practical knowledge and skills, are more likely to engage in teaching and learning activities. The students will be able to perceive the educational potential in their institution. The institution may build multi-level capacities in education, alleviating the increasing demands on senior faculty members.^2^

Although it is not included in the three most popular RaT models (namely, the One-Minute Preceptor, the clinical teaching program of the Stanford Faculty Development Center, and Irby's domains), role-modeling is the most frequently identified method for residents engaged in teaching.^11^

An example of a good technique used in medical education for clinical case presentation is the summarize, narrow, analyze, probe, plan, and select (SNAPPS) technique.^12^ Initially proposed by Wolpaw,^13^ this technique is based on constructive learning wherein students as active participants are able to develop new knowledge and teachers are partners in the learning process.^14^ The use of this technique in the teaching-learning process might help students effectively and efficiently verbalize higher-level thinking skills and improve their technical skills.^12^ In addition, SNAPPS can improve clinical reasoning in the diagnosis and treatment of common diseases^14^ and has the theoretical advantage of placing greater emphasis on self-directed learning.^15^ There have been no previous studies using modified models of SNAPPS for teaching preceptors.

## OBJECTIVE

This paper aims to evaluate whether a 45-minute class using the SNAPPS technique can improve psychiatry residents' case discussion skills in diverse practical learning settings.

## METHODS

This study was conducted in a psychiatric hospital in the city of Fortaleza, Ceará, Brazil, from March 2017 to December 2018. The study included all 27 residents of the psychiatry training program and 15 interns (i.e., medical students from the last two of the six years of medical school in Brazil).

Before data collection, approval from the Research Ethics Board was obtained on 09/01/2017 (No. 2.255.068), and all the participants provided written informed consent.

The study proceeded as follows: Three psychiatric cases were simulated in three different settings: an outpatient clinic, a ward, and the emergency department. The researcher video recorded the 27 residents in the three settings. First, each resident was told to simulate a clinical case supervision with an intern. All the interns received basic instruction lasting around 20 minutes on how to discuss the clinical cases previously prepared by the researchers. They had access to the details of each case on a sheet to better guide the residents as the discussion deepened. Second, they were asked to discuss the cases for up to six minutes. Finally, the cases were provided to the interns to be used when they play the learner-actor role.

**Figure 1:**
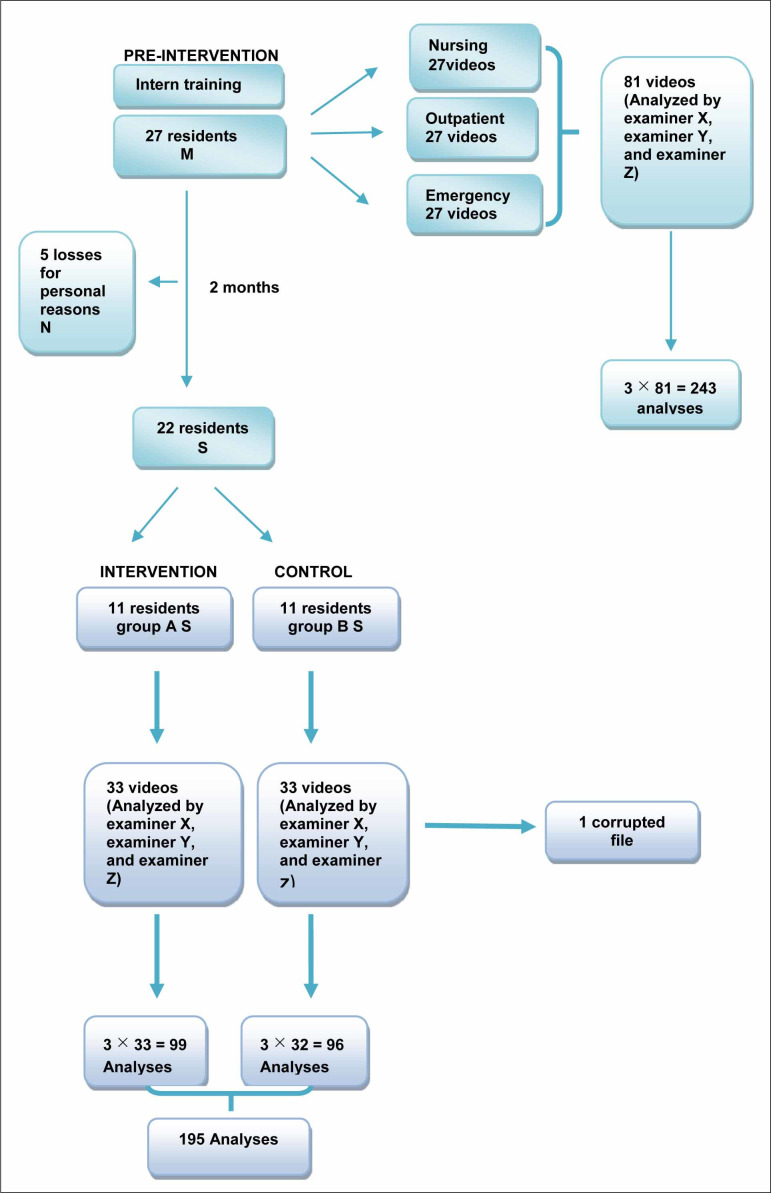
Study design.

Two months after the first phase of the study (pre-intervention), the residents were invited to attend a didactic activity. The residents were randomly assigned to two groups of 11 residents, with each group including equal numbers of first-year (R1), second-year (R2), and third-year residents (R3) (Figure 1). The intervention group (group A) attended a 45-minute lecture on the teaching technique using SNAPPS. Videos of simulated cases were shown, and the residents were taught how to give effective feedback. Contrariwise, the control group (group B) attended a 45-minute lecture on a general topic in the field of psychiatry. Note that unlike the traditional method, only teachers (in this case, the residents) were taught this technique. The interns did not attend the class.

After the lectures, the groups of residents were taken to different places in the hospital and did not meet each other. Thereafter, the residents had another six minutes of discussion in the same three simulated cases and were video recorded again (Figure 1). All the pre- and post-intervention videos were coded, grouped, and recorded. Only 1 out of 144 video files was found to be corrupted and, therefore, could not be evaluated. The videos were analyzed by three blinded assessors who did not have access to the decoding of the study phases. These invited assessors are clinician-educators in another medical school with extensive teaching experience.

After the intervention day, group B residents were invited to attend the 45-minute lecture on the SNAPPS teaching technique. Only one resident did not attend the lecture, for personal reasons.

Each video was assessed three times—outpatient, emergency, and ward—by the three blinded assessors using the Stanford Faculty Development Program (SFDP)-26 tool (validated in Brazil by Fakhouri Filho SA).^16^

A sociodemographic questionnaire was used to collect and assess the residents' gender, year of residency, previous teaching experience, medical school methodology (traditional or active learning), perceived importance of the resident's teaching role, and the approximate amount of time spent teaching during the residency program.

The following analyses were performed: 1) Cronbach's alpha was calculated between the results gathered from the assessors in each stage (pre- and post-intervention); 2) the generalized estimating equation (GEE) approach with gamma distribution and unstructured correlation structure was used to compare the scores in the outpatient, ward, and emergency settings; 3) a chi-squared test or Fisher's exact test (F) was used, as appropriate, for the comparison of demographic characteristics between the two groups of residents. Finally, the Mann–Whitney U test was used for the comparison of variables when there were two groups and the Kruskal–Wallis test where there were more than two. All the tests were performed with a significance level of 5%.

## RESULTS

Internal consistency between external examiners was high in all three settings (outpatient, ward, and emergency), with values of Cronbach's alpha above 0.70. The values obtained in the pre-intervention and post-intervention settings were 0.850 and 0.910 (outpatient), 0.691 and 0.934 (emergency), and 0.701 and 0.885 (nursing), respectively.

An interaction (P < 0.001) in the three settings was observed when comparing the overall score between Group A and Group B. In the outpatient setting, the overall scores ranged from 2.30 ± 0.77 to 6.00 ± 6.76 in Group A and 2.85 ± 0.82 to 2.85 ± 0.78 in Group B. In the emergency setting, they ranged from 2.18 ± 0.82 to 5.06 ± 1.14 in Group A and 2.45 ± 0.85 to 2.36 ± 0.57 in Group B. In the ward setting, the scores ranged from 2.12 ± 0.82 to 5.45 ± 0.67 in Group A and 1.79 ± 0.82 to 2.40 ± 0.81 in Group B.

The analysis of each item separately revealed that some items differed significantly in the outpatient setting (Table 1).

**Table 1:** Mean scores in each item in the outpatient setting

		Group A pre	Group A post	Group B pre	Group B post	Interaction effect	Moment effect	Group effect
**Teaching environment**								
	Wakened students' interest in the topic.	1.24 ± 0.70	4.06 ± 0.71	1.58 ± 0.53	2.18 ± 1.46	P = 0.005	––––	––––
	Encouraged students to actively participate in the discussion.	1.61 ± 1.11	4.85 ± 0.17	2.03 ± 1.52	2.45 ± 1.51	P = 0.008	––––	––––
**Promotion of understanding and retention**								
	Assessed students' level of previous knowledge.	1.21 ± 0.48	3.91 ± 0.91	1.45 ± 1.01	2.18 ± 1.44	P = 0.012	––––	–––––
**Promotion of self-directed learning**								
	Explicitly encouraged further study.	1.00 ± 0.00	4.24 ± 0.56	1.21 ± 0.60	1.21 ± 0.40	P < 0.001	––––	––––
	Politely encouraged students to read while not in the institution.	1.00 ± 0.00	4.42 ± 0.52	1.12 ± 0.31	1.21 ± 0.40	P < 0.001	––––	––––
	Made sure the students understood what was being taught.	1.12 ± 0.31	1.82 ± 0.87	1.03 ± 0.10	1.09 ± 0.30	P = 0.018	––––	––––

The items that differed significantly in the emergency setting are given in Table 2.

**Table 2:** Mean scores in each item in the emergency setting

		Group A pre	Group A post	Group B pre	Group B post	Interaction effect	Moment effect	Group effect
**Promotion of understanding and retention**								
	Assessed students' level of previous knowledge	1.85 ± 1.49	4.21 ± 0.75	1.70 ± 1.39	1.76 ± 0.84	P = 0.029	––––	––––
**Management of the session**								
	Efficiently used the time for teaching	2.42 ± 1.40	4.06 ± 0.59	2.21 ± 1.20	2.06 ± 0.84	P = 0.024	––––	––––

The items that differed significantly in the ward setting are given in Table 3.

**Table 3:** Mean scores in each item in the ward setting

		Group A pre	Group A post	Group B pre	Group B post	Interaction effect	Moment effect	Group effect
**Teaching environment**								
	Awakened students' interest in the topic.	1.73 ± 1.27	4.06 ± 0.96	1.70 ± 1.15	2.00 ± 1.20	P = 0.046	––––	––––
	Encouraged students to actively participate in the discussion.	1.94 ± 1.36	4.48 ± 0.77	1.76 ± 1.15	1.90 ± 1.66	P = 0.048	––––	––––
**Promotion of self-directed learning**								
	Explicitly encouraged further study.	1.33 ± 0.77	4.33 ± 0.80	1.30 ± 1.01	1.70 ± 1.16	P = 0.009	––––	––––
	Politely encouraged students to read while not in the institution.	1.33 ± 0.77	4.33 ± 0.80	1.30 ± 1.01	1.70 ± 1.16	P = 0.009	––––	––––
	Motivated students to study own their own.	1.21 ± 0.48	3.88 ± 1.10	1.30 ± 1.01	1.50 ± 0.97	P = 0.002	––––	––––
**Communicating goals**								
	Presented the expected level of competence.	1.21 ± 0.40	2.97 ± 0.96	1.09 ± 0.30	1.27 ± 0.49	P < 0.001	––––	––––

Comparison of the associations between sociodemographic variables (gender, year of residency, previous teaching experience, time spent teaching, teaching role perceived importance, and medical training methodology) between the two groups—intervention (A) and control (B)—revealed no statistically significant differences (P > 0.05). Most participants (81.8%) were female.

Of the 432 recorded videos, only 1 file was corrupted (Group B in the post-intervention phase in the nursing setting). This loss was discrete and highlighted the statistical data of our study.

## DISCUSSION

In a training program of only 45 minutes, followed by practice, SNAPPS served to consistently improve residents teaching skills. Many studies have also specifically tested and proven the effectiveness of this method in RaT programs.^12,17–22^ Despite their small samples, the results of other studies in psychiatry match the findings of this study, as they demonstrated significant improvements in skills and attitudes.^23–25^

RaT is an easy-to-implement and inexpensive model. Furthermore, unlike traditional methods, our study modified the technique by teaching the preceptors, i.e., the residents. To the best of our knowledge, there are no similar published studies. This is the first study to use a modified model of SNAPPS.

The intervention lasted approximately 45 minutes, which is similar to that in the original SNAPPS study by Wolpaw and other studies,^12,13^ which prevented the activity from becoming tiresome, thereby reducing participant withdrawal rates as video recordings took place in different shifts. No particular mode or duration of RaT programs can be considered better than others. The programs may include simple lectures, teaching retreats lasting several days, didactic classes, and even online modules.^3,4^

Similar to the study by Connor, the SNAPPS technique was also evaluated shortly after the lecture.^20^ As in other studies,^26^ the residents were independently assessed by three blinded assessors. Reliability was guaranteed by the standardization of the assessment, which allowed the external examiners to assess the residents with high internal consistency.

The SNAPPS technique had a positive impact on the residents. It improved their skills in managing a case discussion session with interns. The individual items specifically related to awakening the interest of interns in the topic, encouraging their active participation, and assessing their level of previous knowledge were found to be significantly different between Group A and Group B. These items refer to primordial skills taught in the SNAPPS lecture that were properly learned and put into practice by the residents.

Items such as listening carefully to the students (interns), showing respect, not ridiculing them, and answering their questions clearly and politely did not present any interaction effects in any of the three settings. Those attitudes are probably already part of the residents' behavior in psychiatry and may have been acquired throughout life or properly modeled during undergraduate studies.

The sociodemographic variables did not differ significantly between Group A and Group B. Third-year residents have similar teaching skills as first-year residents. Our findings suggest that, without proper training, the residents did not necessarily improve their teaching skills regardless of their year of residency. They need specific training to acquire such skills. Similar results were reported by Sawanyawisuth et al.,^18^ in which the differences found in the SNAPPS group resulted due to maturation over time, as fifth-year students performed better than sixth-year students on basic attributes, having more diagnoses in their differential, more justified diagnoses, and initiating more diagnosis.^18^

When asked about the amount of time spent teaching in medical residency, most residents (25 out of 27) reported spending 25% of their time teaching interns or fellow residents. Isenberg-Grzeda et al. ^27^ found that 86% of respondents reported that teaching is a common activity during a typical week. In another study, 50% of the residents reported teaching daily, 40% reported teaching only a few times a week, and 10% reported teaching a few times a month.^28^

With regard to the importance of residents as teachers, only 1 of the 27 participants did not find this role important. While it was not possible to attest this statistically, it is clear that the subject is of great importance to residents and to interns, who usually start learning from the residents shortly before starting residency. Similar data have been found in a study that reported that most participants (87%) found teaching to be pleasant or rewarding, 79% wished to continue teaching after residency, and 72% believed that RaT programs should be mandatory.^27^ These findings are also supported by a study of psychiatry residents that reported a score of 4.53 out of 5 for the item “I think teaching medical students is an important role of residents.”^29^ The residents who had experienced active teaching methodologies during their undergraduate studies were expected to present better scores than those who had learned from traditional teaching methods. However, the scores were practically the same in the three settings, and there were no statistically significant differences.

This is the first study to use the SNAPPS teaching technique in Brazil. It tested the technique only on teachers (residents) and found statistically significant results in three different settings; moreover, it found the residents' interest and willingness to participate to be quite significant.

Although there were many assessments of the residents due to the analysis of three settings by three examiners, the number of residents who participated in this study was relatively small and were drawn from only one medical specialty, thus impairing the generalizability of the results. Furthermore, we did not reassess the residents' performance a few months after the intervention, which would be highly useful for evaluating the retention and effectiveness of the method applied. Further, the interns' perceptions as actors were not assessed nor those of the residents of their role as clinician-educators.

Medical education has undergone an important and substantial evolution since last century. Frenk identified and described three phases of this evolution, as shown in the Chart 1.^30^

**Chart 1:** Medical education evolution

A global review identified the following three phases in the evolution of medical education: (1) A formative phase characterized by didactic teaching, phenomenological and memory learning, and a focus on the scientific basis for medicine during the first 70 years of the 20th century; (2) A performative phase characterized by problem-based instructional innovations focused on concepts in biology as applied to medicine, data retrieval, and integration of knowledge during the latter decades of the 20th century; (3) A transformative phase starting in the 21st century to improve the performance of health systems by adapting core professional competencies to specific contexts while drawing on global knowledge.^30^

Adapted from Frenk et al.^30^

Current evidence suggests that some active learning methodologies show a significant improvement in student learning over traditional teaching methods. Meta-analyses of flipped classrooms,^31^ team-based learning (TBL),^32^ simulation-based medical education (SBME)^33^ with deliberate practice (DP), and problem-based learning (PBL)^34^ seem to be more effective in improving students' knowledge, attitudes, and skills. There has been no meta-analysis comparing RAT or SNAPPS to traditional training methods.

Recently, we have achieved an improvement of medical education by the implementation of new types of learning such as e-learning (since the emergence of the Internet) and blended learning that show significantly better knowledge outcomes than those for traditional learning, as shown in the meta-analysis by Vallée in 2020.^35^ They can transcend the previous restrictions of space and time as well as improve collaborative and individualized learning effectiveness.^35^

As a promising tool for medical learning in the future, Free Open Access Med(ical edu)cation, FOAMed is a dynamic collection of articles, apps, and audio and video materials produced to support clinicians' lifelong learning. It began in emergency medicine (EM) but has spread to critical care (CC), pediatrics, and toxicology to become a large repository of Internet-based resources provided by a large social media community as a means of delivering high-quality medical education to anyone with a device.^36^

Some advantages of PBL were described in the study by Jones in 2006, and can illustrate the benefits of many other active learning methodologies: facilitating trainees in becoming responsible for their own learning, making curriculum content relevant by building learning around clinical, community, or scientific problems, and increasing the motivation of trainees to learn by focusing the learning on “real-life” scenarios.^34^ In practical learning settings, preceptors' view of the traditional presentation identify generic skills such as history-taking and presentation skills. Lack of time and objective feedback is also recognized as a deficit of traditional clinical training.^37^

Disadvantages of new learning methods can also be pointed out: The knowledge acquired through PBL is less organized than that acquired through traditional learning; more time is required of trainees to fully engage in new learning methods; the replacement of the traditional teacher role by the facilitator may make it difficult for trainees to emulate good teachers as role models; and significant costs, resources, and time are required to train effective facilitators.^34^

## CONCLUSION

Generally, the SNAPPS group had significantly higher scores than the traditional case presentation group in the outpatient, ward, and emergency settings, as assessed by OSTE using SFDP-26. There were no correlations of the results with sociodemographic variables, such as gender, year of residency, previous experience in teaching, or undergraduate medical school methodology.

Despite being targeted only at residents who performed teaching functions, the lecture on the SNAPPS technique has proven effective and can be useful in medical teaching for the improvement of skill acquisition. As in peer learning, where the use of two-way processes and reciprocal learning activities is important, SNAPPS involves the sharing of knowledge, ideas, and experience among participants for mutual learning in undergraduate medical schools. This type of activity can have an impact on medical practice in Brazil and other countries, where studies on RaT and OSTE are still emerging. If implemented systematically as part of an RaT program, the residency will benefit from an approach that can improve the teaching of clinical reasoning. Further studies using SNAPPS and other case presentation techniques are needed to consolidate such active teaching methodologies. Pedagogical surveys to identify residents' opinions about the method are also important.
